# Leveraging the Sensitivity of Plants with Deep Learning to Recognize Human Emotions

**DOI:** 10.3390/s24061917

**Published:** 2024-03-16

**Authors:** Jakob Adrian Kruse, Leon Ciechanowski, Ambre Dupuis, Ignacio Vazquez, Peter A. Gloor

**Affiliations:** 1 School of Computation, Information and Technology, Technische Universität München (TUM), Arcisstr. 21, 80333 München, Germany; jakobk@mit.edu; 2 MIT Center for Collective Intelligence, 245 First St., E94-1509, Cambridge, MA 02142, USA; lciechanowski@kozminski.edu.pl (L.C.); ambre-manon.dupuis@polymtl.ca (A.D.); 3 Department of Management in the Network Society, Kozminski University, Jagiellonska 57, 03-301 Warszawa, Poland; 4 Laboratoire en Intelligence des Données (LID), École Polytechnique de Montréal, CP 6079, Succursale Centre-Ville, Montréal, QC H3C 3A7, Canada; 5 MIT System Design & Management, 21 Amherst St., E40-338, Cambridge, MA 02142, USA; ignaciov@mit.edu

**Keywords:** emotion recognition, artificial intelligence, deep learning, plant sensor, classification, emotion models

## Abstract

Recent advances in artificial intelligence combined with behavioral sciences have led to the development of cutting-edge tools for recognizing human emotions based on text, video, audio, and physiological data. However, these data sources are expensive, intrusive, and regulated, unlike plants, which have been shown to be sensitive to human steps and sounds. A methodology to use plants as human emotion detectors is proposed. Electrical signals from plants were tracked and labeled based on video data. The labeled data were then used for classification., and the MLP, biLSTM, MFCC-CNN, MFCC-ResNet, Random Forest, 1-Dimensional CNN, and biLSTM (without windowing) models were set using a grid search algorithm with cross-validation. Finally, the best-parameterized models were trained and used on the test set for classification. The performance of this methodology was measured via a case study with 54 participants who were watching an emotionally charged video; as ground truth, their facial emotions were simultaneously measured using facial emotion analysis. The Random Forest model shows the best performance, particularly in recognizing high-arousal emotions, achieving an overall weighted accuracy of 55.2% and demonstrating high weighted recall in emotions such as fear (61.0%) and happiness (60.4%). The MFCC-ResNet model offers decently balanced results, with $Accuracy_{MFCC-ResNet}=0.318$ and $Recall_{MFCC-ResNet}=0.324$. Regarding the MFCC-ResNet model, fear and anger were recognized with 75% and 50% recall, respectively. Thus, using plants as an emotion recognition tool seems worth investigating, addressing both cost and privacy concerns.

## 1. Introduction

Emotions are an integral part of the human being. They condition our actions and decisions [1]. There’s nothing more personal than an emotion. Ekman and Friesen [2], however, have demonstrated the universal nature of basic emotions, enabling everyone to recognize them, independently of their culture or education.

Human emotion recognition is a widely studied topic in human behavioral research. Different types of data such as video [3,4,5], speech [6,7], and text [8,9,10] are used for analysis. However, the high cost (in terms of acquisition, operation, and maintenance), as well as the privacy intrusiveness and regulations surrounding these types of data, are obstacles to the development of human emotion detection [11]. Unlike these sensors, which are difficult to accept, plants are part of people’s daily lives. In addition to their benefits for the quality of human life [12], it has been shown that plants can sense human steps [11] and sounds [13]. The question then arises of whether this ability of plants can be leveraged to detect human emotions.

This article proposes a methodology using plants as sensors to detect human emotions. This approach addresses the concerns and high costs of traditional emotion-tracking methods and will pave the way for new research in human emotion detection and recognition.

The article is structured as follows. Section 2 provides an overview of the research on human emotion detection as well as the use of plants as sensors. Then, Section 3 presents the material and methodology necessary to use plants as human emotion detectors. Section 4 will describe the results obtained by applying the proposed methodology to a real-world experiment. The limitations and directions for further research are discussed in Section 5. Finally, Section 6 concludes the article by recalling the contributions and limits of the proposed methodology, as well as future research directions.

## 2. State of the Art

Although both plants and emotions are an integral part of people’s daily lives, the use of the former to recognize the latter is unprecedented. In order to better understand the foundations supporting the development of a methodology enabling the use of plants as human emotion detectors, an overview of the research on emotion recognition (Section 2.1) and plant-based sensors (Section 2.2) is given in the following section.

### 2.1. Emotion Recognition

**Figure 1:**
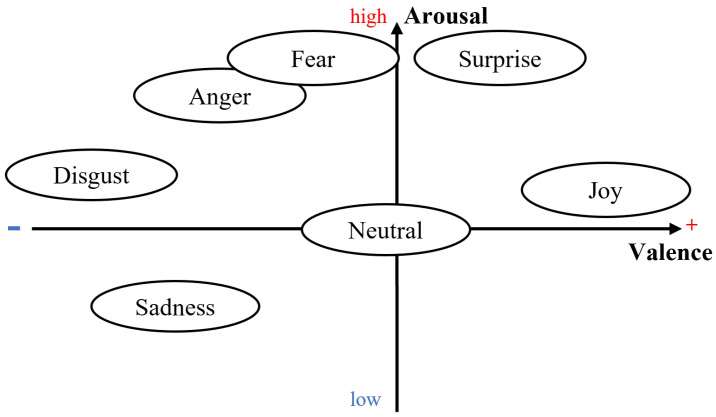
Approximate position of the Ekman [19] basic emotions on the Russell [20] valence-arousal model.

What could be more personal than an emotion? Emotions reflect an automatic, unconscious evaluation of a situation based on past personal experience and human evolution [14]. Thus, emotions are, by definition, subjective and personal. Since each person has their own personal history, the same situation can provoke a wide variety of emotions of varying intensity in different people [15]. Despite the various psychological and philosophical debates on the rational (cognitive and intellectual) or irrational (emotional and social) nature of human beings [16], the power and omnipresence of emotions, as well as their influence on decision-making, is widely accepted [1]. Emotions have a major influence on an individual’s reactions (choices and actions) and on the assessment of others’ behavior in a given situation [15]. The structure of emotional spaces is still up for debate [17]. Since Darwin [18] first introduced the notion of emotions, different approaches have been proposed. For Ekman [19], six basic emotions are present in all cultures and are recognizable by the same facial expressions. This supports the universalist theory of emotions to the detriment of the cultural hypothesis [2,14]. Thus, for Ekman [19], fear, anger, joy, surprise, disgust, and sadness are the primary colors of the chromatic palette of emotions. When the basic emotions are mixed, they form a vast variety of more complex emotions, such as pride, excitement, or amusement [14]. Others, such as Russell [20], model emotions on a continuum. The valence-arousal model describes a person’s emotional state according to the level of pleasantness (valence positive or negative), and arousal (arousal high or low) of the emotion felt [20]. These two models are not mutually exclusive. Indeed, the six basic emotions defined in [19] and the resulting complex emotions can be placed on the continuum proposed in [20], as illustrated in Figure 1.

With the advancement of new technologies and the constant increase in computing power, these conceptualizations of human emotion form the basis of automatic emotion recognition (AER). Although the discrete conceptualization of emotions (such as Ekman [19]) results in a classification problem and is cited frequently in the relevant literature [17], the **valence-arousal** continuum can also be used for regression purposes [4,5]. Regardless of the chosen framework, a wide range of data sources can be used to detect emotions. When leveraging the most recent advances in AI and deep learning, the main input sources for AER are natural language processing (NLP) for text, facial emotion recognition, and speech emotion recognition.

Text analysis is one of the most widely used techniques due to the abundance of opinions and emotions shared by individuals on social networks or ecommerce platforms [9,10]. Emotion detection from text can be handled by a keyword-based approach or, more rarely, a rule-based approach. However, the most commonly used approach is based on learning since it offers the best performance when dealing with the implicit expression of emotions (such as irony or sarcasm) [9,10]. Within this approach, we find machine learning tools (support vector machine (SVM), decision trees, random forest (RF), k-NN, and the hidden Markov model (HMM) [8]) and deep learning tools (convolutional neural networks (CNNs), long short-term memory (LSTM), and transformers) [9,10]. SVM, CNN, and LSTM are also commonly used for emotion detection using image analysis [3].

Image analysis is extremely relevant for emotion detection since two-thirds of the information transmitted by an individual is in the form of non-verbal elements, particularly through facial expressions [3]. The work of Ekman and Friesen [21] makes it possible to characterize the basic emotions described by Ekman [19], which are felt by an individual and are represented by facial expressions. More recently, the hand-crafted attributes used in conventional methods have been replaced by training automated facial emotion recognition (FER) models, such as in the work of Mühler [22], which automatically generate attributes for classification [3,23]. Although non-verbal information is omnipresent, speech remains the most natural and fastest method of communication for humans. Speech analysis is, therefore, an important element of AER.

Speech analysis uses the same classification tools as image or text analysis. These include machine learning methods, such as SVM, HMM, or k-NN [6], but also deep learning approaches, such as DNNs, CNNs, and LSTM [7]. One of the most important challenges faced in speech analysis is the extraction of attributes with significant emotional representativeness, regardless of the lexical content used. El Ayadi et al. [6] describe continuous, qualitative, spectral, and Teager energy operator (TEO)-based attributes as the four categories of attributes that can be extracted from speech analysis. Spectral attributes are used as a short-term representation of speech signals, and Mel frequency cepstral coefficient (MFCC) feature extraction is an important data-processing tool since these features hold promise for the representation of speech in multiclass classification [6]. Although many advances have been made in the use of text, images, and speech for emotion recognition, they are based on elements that can be consciously manipulated by individuals. Physiological data can limit that risk.

The expression of emotions generates physiological changes in individuals that are hard to fake [24]. Thus, physiological data, such as brain activity (EEG), body temperature, heart rate signals (ECG), muscle activity (EMG), sweat levels, and respiration levels, can be used as “honest” input into emotion recognition models [24]. Once again, machine learning and deep learning tools, such as SVM, k-NN, RF, CNN, LSTM, and DNN, are used in this context.

The influence of emotions on people’s choices and behavior makes AER an exciting field of study for many sectors, such as robotics, marketing, education, healthcare [9,10], and also finance [8]. For an in-depth discussion of AER, see the work of Kruse [25].

However, the high cost (in terms of acquisition, operation, and maintenance), as well as privacy intrusions and regulatory restrictions when collecting these types of data (video, audio, text, EEG, etc.), are an obstacle to the development of automated human emotion detection systems [11].

### 2.2. Using Plants as Sensors

Unlike cameras, microphones, or EEG, plants are part of people’s daily lives. In addition to their benefits for the quality of human life [12], it has been shown that plants can be used as sensors that are able to monitor their environment [26]. More precisely, plants are sensitive to changes in luminous intensity, pressure, and temperature, as well as changes in the electromagnetic or gravitational fields [27]. They respond to these environmental changes by generating different types of electrical signals within and between cells [26]. The effects of environmental changes on plants can be rapidly observed thanks to the propagation speed of bioelectric signals, ranging from 0.05 cm/s to 40 m/s [27]. In order to detect electrical reactions in plants, electrodes can be placed on the plant [28] or directly inside the plant, in contact with the targeted cells [29]. The resulting signal corresponds to the difference in potential between the plant cells and the soil. The electrical analysis of plants has been carried out for many plant species; for instance, Oezkaya and Gloor [11] used *Mimosa pudica* for their experiments, whereas Peter [13] used *Codariocalyx motorius*. Frequently, basil (*Ocimum basilicum*) is used, as its leaves are sensitive to the electrodes, and it is easily available in local supermarkets.

The use of electrical signals emanating from plants as sensors has been exploited in a limited number of works. Chatterjee et al. [30] used electrical signals from tomato plants to recognize the chemicals that the plants are exposed to. Signals collected using internal electrodes placed in roots, stems, and leaves are statistically processed, and the extracted attributes are used as input to a linear discriminant analysis (LDA) classification model. The study classifies the sodium chloride (NaCl), sulphuric acid (H2SO4), and ozone (O3) to which plants are exposed, with an accuracy of approximately 70%.

In addition to their ability to recognize chemicals, plants can also be useful for recognizing individuals and their moods (happy or sad) [11]. Oezkaya and Gloor [11] used external electrodes with the *SpikerBox* [31] to record electrostatic changes caused by a person’s gait. The signal generated by the plant is processed by MFCC and then classified using a random forest (RF) model. By using this method, one can recognize one individual among six others with an accuracy of 66%. It can also determine a person’s mood with an accuracy of 85%. Finally, Peter [13] showed that plants are also sensitive to sound. Using statistical modeling of plant signals combined with an MLP classifier, the three sounds used in the experiments were classified with an accuracy of 72%.

Thus, it has been shown that the external sensing capabilities of plants can be used for the development of sensors with a quality comparable to that of dedicated devices. With simple, inexpensive operations and no need to record personal data, plants appear to represent an interesting data-acquisition tool that remains relatively unexploited. In the remainder of this paper, we investigate the ability of plants to detect human emotions.

## 3. Method

Traditional sensors to capture human emotions are often perceived as intrusive; they are highly regulated and can be costly to acquire, operate, and maintain. On the other hand, plants are an integral part of our environment. In addition to their aesthetic aspects, it has been shown that plants are endowed with an astonishing capability for sensing their environment [11,13,30]. This study seeks to further this knowledge and proposes a methodology for enabling the use of plants as human emotion detectors. As has been mentioned above, earlier work [11] has shown the promise of plants to be used as sensors to recognize the positive and negative moods of humans by measuring electrical changes in the voltage between the roots and leaves of a plant. In this research, we investigate if a plant is capable of identifying more granular emotions, distinguishing between the six Ekman emotions [19]. The main challenges of this work were two-fold: first, an apparatus needed to be developed capable of measuring these potential differences of the plant at the millivolt level. Secondly, a machine learning model needed to be developed, which could use these voltage change time series as input and predict the emotions of humans near the plant based on these voltage changes. In order to test this research hypothesis, a method was developed and verified in an experiment.

### 3.1. Experimental Setup

**Figure 2:**
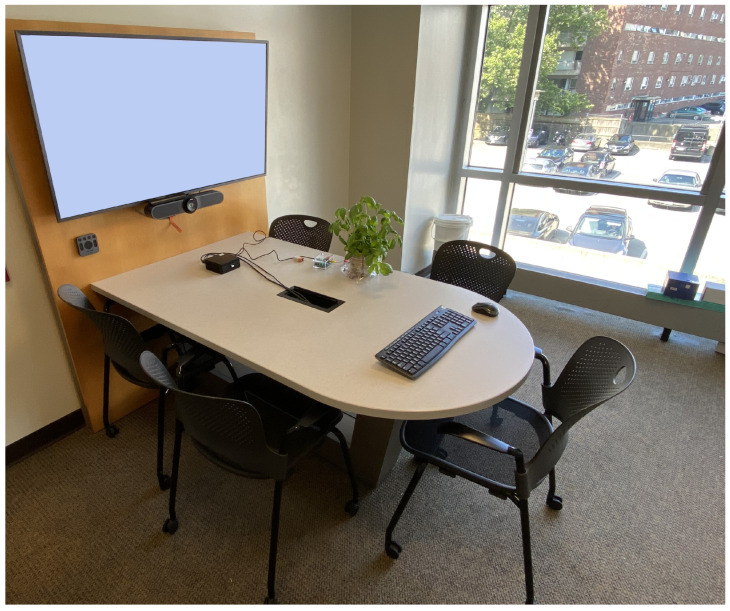
Experimental setup to detect human emotions with a plant-based sensor.

In order to develop a methodology to detect human emotions using a plant, an experiment was designed to simultaneously collect the emotions of an individual and the reactions of a plant to the human [25]. The purpose of the experiment was to induce strong emotions in the participant and to record the responses obtained by the plant as a sensor. The experiment was structured into five steps:

1. First, the participant’s consent was collected, and the observer in charge of running the session answered any open questions.
2. Then, the observer quickly described the task that the participant would be asked to perform. The task was to watch a video sequence designed to elicit strong emotional responses from participants. The video sequence was created based on previous work by Gloor et al. [32], and the details of the video are described in Table 1.
3. The participant then sat in the experimental room, and the sensors (plant and camera) were activated. Figure 2 is a photograph of the experimental setup.
  A screen displayed the videos to elicit the participants’ emotional reactions (see Table 1). These reactions were filmed by a wide-angle Logitech Meetup camera placed just below the screen. The camera was set up to obtain a zoomed image of the participant’s face. Additionally, the participants each wore a wired headset microphone connected to a hand-held voice recorder that was used to record their voices. The camera also recorded the voice and provided an additional backup data source for this modality. Finally, a basil plant, *Ocimum basilicum*, equipped with a sensor, *SpikerBox* [31], was positioned in front of the participant.
4. Then, the observer started the video sequence and left the room to allow the participant to watch the video.
5. Once the video sequence was finished, all sensors were deactivated, and the data collected by the plant sensor and the camera were saved.

This protocol was repeated for each participant in the study. As the participants watched the videos, their emotional reactions were recorded by the plant-based sensor and the camera. The individual files were then stored in two databases called “plant signals” and “video”.

### 3.2. Analysis

**Figure 3:**
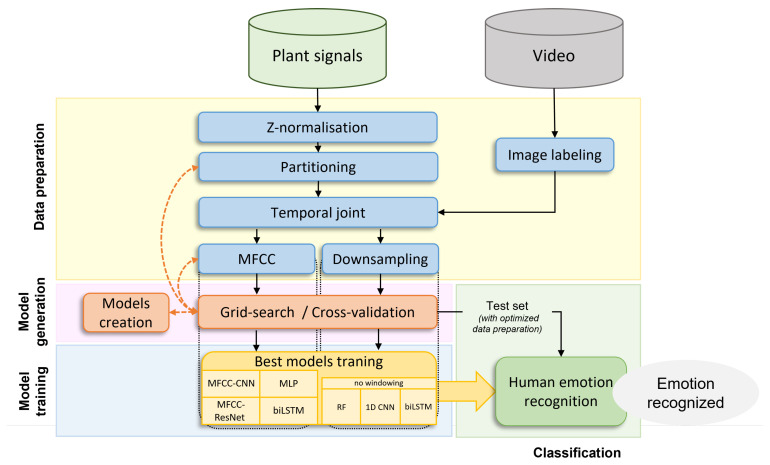
Four-step algorithm to detect human emotions with a plant-based sensor.

In order to analyze the data, the four-step algorithm illustrated in Figure 3 was used.

First, the data were preprocessed. The plant signals were cleaned and formatted so that the classification algorithms could use them. They were also labeled using video data, which computed the emotions felt by the participants during each second of the experiment from their facial expressions. Once the plant signals were formatted and labeled, they were used in the subsequent classification model generation step.

In the classification model generation step, different deep learning models were tested and parameterized using the cross-validation and grid-search algorithms. The grid-search algorithm used the cross-validation folders to find the best combination of hyperparameters for both model architecture and data preparation. The best parametrization for each of the models was saved and used in the model training step.

In the model training step, training and validation sets were once again used to train the selected models further. The trained models were finally tested with the test set. The goal was for the plant sensor to exclusively predict the emotion of the viewer.

Each step of the methodology is detailed below.

### 3.3. Data Preparation

Data preparation is a necessary step to transform raw data into data that can be used by artificial intelligence algorithms. Although both data sources needed to be pre-processed, the signals from the plant sensor underwent more significant transformations. In our research, we implemented two distinct methodologies for data preprocessing, each yielding divergent outcomes. The initial method, henceforth referred to as “MFCC Extraction”, involves segmenting the electrical signal into 20 s intervals, followed by the extraction of Mel Frequency Cepstral Coefficients (MFCCs) from these segments. The second method, which we designate as “Raw Signal Analysis”, entails isolating 1 s fragments from the raw electrical signal. These fragments directly correspond to the facial emotions detected over the duration of 1 s. This approach focuses on analyzing the amplitude variations within the signal. The methodologies are described in detail in the subsequent sections.

#### 3.3.1. Initial Data Preparation Approach—MFCC Extraction with Windowing

First, each signal needed to be treated in order to limit the effect of the experimental conditions on signal analysis. Indeed, although the experimental conditions were controlled, interferences due to the environment or the position of the sensors on the plant can alter the signal. These conditions are considered constant throughout the same experimental session. The signals were normalized using z-normalization on a per-file basis.

Then, the signal was partitioned in order to transform it into a set of shorter windows. Each window represents a portion of the normalized signal, the length of which is determined by the hyperparameter *window*, expressed in seconds. Another hyperparameter named *hop* allows one to choose the number of seconds between the beginning of two successive windows. This specificity allows for the succession of overlapping time windows if the *hop* value is smaller than the *window* size. This is the principle of the sliding window that is used in time series processing. The hyperparameters were tuned using the cross-validation and grid-search algorithm presented in the following step of the methodology.

In order to classify the responses of the plant according to the emotions felt by the participants, it was important to collect the real emotion felt by them. Image analysis for emotion detection has been widely studied and recognized by the scientific community (see Section 2.1). Thus, the emotions recognized in the images extracted from the videos are used as ground truth in order to label the signals obtained by the plant-based sensor. During image labeling, each second of the video results in the extraction of an image. Those images are then used as input to the well-known emotion detection model *face-api.js* [22] to obtain the emotion felt by the participant during the one-second window considered. Face-api.js offers robust face detection capabilities, primarily through its most accurate single shot multibox detector (SSD)-based model, which integrates the lightweight MobileNet V1 CNN with additional layers for box predictions. This setup has been recognized for its high efficacy in face detection and face emotion recognition tasks, reporting accuracy rates of between 86% [33] and 90% [34] in various studies. Such performance underscores the model’s potential for reliable emotion detection, making it a credible choice for establishing ground truth in emotion recognition research.

A temporal join is then used to associate the labels obtained from the video input with the processed signals obtained from the plant sensor. The plant data window labels are assigned based on the label at the end of the data window. Furthermore, those data points where the label proposed by face-api.js does not match the expected emotion specified in the column **Expected emotion** of Table 1 were excluded. The emotions to be detected are stored in an emotion list containing $nb_{emotion}$ different emotions.

Finally, two independent datasets were created from the short plant signals.

- The first dataset consists of the downsampled short plant signals. Since the plant sensor has a high sampling rate (10 kHz), a downsampling of the signals is required before feeding them into the different training models. Downsampling reduces the complexity of the signal while retaining the relevant information [11]. Its rate is a hyperparameter called *downsampling rate*, the value of which is determined by trial and error.
- The second dataset consists of the computation of the MFCC features from each window. The result is a 2D matrix [*time steps*; *number of MFCCs*] that can be processed by various deep learning algorithms, such as LSTM or CNN.

Obtaining these two datasets concludes the first step of the methodology: data processing. The classification models as well as the optimization of the hyperparameters can be initiated in the second step of the methodology: model generation.

#### 3.3.2. Alternative Data Preparation Approach—Raw Electrical Signal Analysis

In contrast to the previous approach, the alternative data preparation method focuses on analyzing the raw electrical signals from the plant sensor without downsampling and windowing. This approach aims to explore the full complexity and granularity of the data, potentially revealing subtle nuances in the signals that are associated with different emotional responses in participants.

The electrical signals are captured from a *SpikerBox* [31] attached to a plant in the same environment as participants viewing emotional videos. Each participant’s electrical signal data, represented as an array, consists of 6,900,000 samples, reflecting the sampling rate of the sensor (10,000 Hz) and the total length of the watched videos (690 s). The ground truth for the emotions was derived from video recordings labeled with timestamps and the corresponding emotions.

The raw electrical signals were processed without downsampling to preserve the fidelity of the original signal. The signal for each participant is segmented into 1 s intervals, corresponding to the timestamps of the emotion data. This segmentation results in 690 segments per participant, each containing 10,000 samples. Each signal segment was normalized using z-normalization to reduce the impact of any variations in signal amplitude and to facilitate comparability across the different participants. Instead of downsampling or computing the MFCC features, this approach focuses on analyzing the raw, normalized signal data. This decision is based on the hypothesis that the high-resolution data might contain intricate patterns associated with different emotional states.

The labeled emotions from the video data were used to tag each corresponding signal segment. The process involved aligning the timestamps from the emotion data with the signal segments, ensuring that each segment was labeled with the corresponding emotional state of the participant at that specific second. The normalized signal segments and their associated emotion labels were integrated into a cohesive dataset. This dataset forms the basis for the subsequent modeling and analysis, wherein the relationship between the raw electrical signals and the participants’ emotions was explored.

The final dataset comprises two main components: the normalized electrical signal segments and their corresponding emotion labels. This dataset was taken into the next phase of the study, which involved the development and training of the models to classify the signals based on the emotional states of the participants.

### 3.4. Model Generation

The generation of classification models allows for the definition of their architecture and also their parameterization. Many hyperparameters influence the performance of the studied models, which is why it is important to choose them carefully.

The creation of the models aims to define the general architecture of the different models considered for the detection of human emotions from plant signals. This task corresponds to a multiclass classification of a time series. As neural networks are known to perform well in these tasks (see Section 2.1), three different types of architectures were considered. Table 2 summarizes the key elements of the architecture of each model. All models produced an output of a vector of size $\left[1,nb_{emotion}\right]$, representing the probability of occurrence of each detected emotion present in the emotions list.

A fully connected multi-layer perceptron neural network (**MLP**) was used as a baseline, and LSTM-based models (**biLSTM**), CNN-based models (**MFCC-CNN**, and **MFCC-ResNet**) were used to process the downsampled signals, and MFCC features, respectively. **ResNet** is a deeper convolutional network proposed by He et al. [35] and is trained on ImageNet [36]. The model **MFCC-ResNet** uses a neural network **ResNet** to process the MFCC data extracted during the data preparation phase. In this work, the architecture of the neural network **ResNet** was slightly modified to fit the emotion detection task. Indeed, the last layer of every model is expected to be a dense layer that is activated by the SoftMax activation function, containing $nb_{emotion}$ neurons, each representing one of the detected emotions present in the Emotions list. Additionally, for the raw, unwindowed plant electrical data, a random forest model was employed, taking advantage of its ability to handle diverse datasets efficiently. It operates using a large ensemble of decision trees, configured with parameters such as the number of trees and maximum depth to ensure a balance in managing complex classification tasks. A one-dimensional CNN (1D CNN), which is particularly suited to analyzing time series data, was also utilized for handling the raw plant electrical signals. This model consists of a sequence of convolutional and pooling layers, followed by dense layers with a ‘SoftMax’ activation function in the output layer for class probability estimation. Lastly, another variant of the LSTM model, the biLSTM, was applied to capture the long-term dependencies in the time-series data. This model’s architecture features bi-directional LSTM layers followed by dense layers, fine-tuned to optimize performance in emotion detection tasks. All models share the common trait of outputting a probability vector, indicating the likelihood of each emotion detected in the dataset. The output vector of all models is then a probability vector of size [1,$nb_{emotion}$].

Each of the models has hyperparameters; it is important that these hyperparameters are optimized to ensure the best performance. Table 3 presents the specific values associated with each of the models’ hyperparameters, which should be tested during the grid search.

In addition to the hyperparameters specific to each model, the general hyperparameters related to data preparation or model training also needed to be taken into account. Table 3 summarizes all these hyperparameters and defines the ranges of values to be tested during the optimization of hyperparameters by a grid search.

In all models considered, the optimization of the hyperparameters *window* and *hop* used in the partitioning task during the data preparation was needed. This is also the case for the *learning rate* and for *balancing* hyperparameters. The learning rate is used for the training of each model, whereas the *balancing* hyperparameter is used to handle the unbalanced dataset. When the value *balance* is chosen, rare classes are oversampled, whereas the majority classes are undersampled. In the case where the value *weights* is chosen, the weight of each class is incorporated into the loss function so that all classes have the same impact on this loss. Finally, if the value *none* is chosen, nothing is enacted to compensate for the data unbalancing.

The other hyperparameters are model-specific. In the **MLP model**, the number of dense layers (*dense layers*), the number of neurons per layer (*dense units*), and the dropout rate (*dropout rate*) are the hyperparameters inherent to the model. Similarly, in the **biLSTM model**, the number of LSTM layers (*LSTM Layers*), the number of neurons per layer (*LSTM units*), and the dropout rate (*dropout rate*) are the hyperparameters with which to optimize it. In the **MFCC-CNN model**, the *dropout rate*, the number of convolutional layers (*conv layers*), the output dimensionality (*conv filter*), and the size of the 2D convolution window (*conv kernel size*) are the hyperparameters inherent to the model. Finally, In the **MFCC-ResNet model**, only two hyperparameters are model-specific. The *pretrained* hyperparameter determines if the weights from the ImageNet training were used, whereas the *number of MFCCs* determines the number of MFCC features extracted during the data preparation step. In the case of the **Random Forest** model, the key hyperparameters include the number of trees in the forest (*n_estimators*), the maximum depth of the trees (*max_depth*), and the class weights (*class_weight*). For the **1D CNN** model, the important hyperparameters comprise the number of filters (*filters*), the kernel size (*kernel_size*) in the convolutional layers, the pool size (*pool_size*) in the pooling layers, and the number of neurons in the dense layers (*dense units*). The model is also characterized by its learning rate (*learning_rate*) and loss function (*loss*). Lastly, in the **biLSTM** model, the critical hyperparameters include the number of LSTM layers (*LSTM Layers*), the number of units in each LSTM layer (*LSTM units*), and the learning rate (*learning_rate*) of the optimizer. These hyperparameters are crucial for fine-tuning the models’ performance in emotion-detection tasks.

The combinations of each value presented in Table 3 were tested by training each classification model independently over a predetermined number of epochs.

### 3.5. Model Training

In order to identify the best parameters for each model, a grid search of the configuration was used with cross-validation. A five-fold cross-validation leads to a training, validation, and test split of 60%, 20%, and 20% of the data.

Since the output of all presented models is a probability vector obtained from the SoftMax activation function, the standard associated loss function called categorical cross-entropy (CE) is used for training [37]. As explained by Qin et al. [37], the measurement of the cross-entropy between the true label *y* and the label *ŷ* resulting from the SoftMax activation function allows for adjusting the model parameters by backpropagation. The stochastic gradient descent method known as the Adam optimizer [38] was used to train the models on the training data, grouped by batch sizes of 64. The overfitting of the model was monitored using the validation set.

Two metrics were used to measure the performance of the models during training. The overall accuracy presented in Equation (1) measures the rate of the correct prediction of the model. The average recall per class presented in Equation (2) measures the sensibility of the model. In other words, it measures the ability of the model to find a good positive class.

(1) $$Overall\;Accuracy=\frac{TP+TN}{TP+FP+TN+FN}$$

(2) $$Average\;Recall\;per\;Class=\frac{1}{nb_{class}}\sum_{\forall class}Recall_{class}=\frac{1}{nb_{class}}\sum_{\forall class}{\left(\frac{TP}{TP+FN}\right)}_{class}$$

where

$$\begin{array}{cc} \; & TP\;=\;True\;Positive\\ \; & FP\;=\;False\;Positive\\ \; & TN\;=\;True\;Negative\\ \; & FN\;=\;False\;Negative \end{array}$$

During the grid search, each configuration is tested with the training and validation sets of different folds. Thus, the whole dataset is found at least once in the test. The models are trained on a limited number of epochs. By using the *EarlyStopping* function, the training can be stopped if the loss on the validation set does not improve after a predetermined number of epochs, which is called *patience*. The performance of each configuration is the average of the metrics (overall accuracy and recall) obtained for each fold.

Once this first training is finished, the configuration having obtained the best score for each model was kept and was used for the final training.

The final training is exactly the same as the one used during the grid search, with the difference being that the number of training periods is higher.

These can now be used in the last step: classification.

### 3.6. Classification

The classification step aims to feed the trained models with new data that they have never observed before (test set) in order to detect the emotion associated with the input signal. As was explained in Section 3.4, the vector obtained in the output of the model is a vector of size [$1,nb_{emotion}$], representing the probability of each of the detected emotions according to the input signal used. The emotion recognized by the model from the input signal corresponds to the class associated with the highest probability in the resulting output vector of the model. The classification performance of the models is evaluated in the case study presented below (Section 4).

## 4. Results

The methodology described in Section 3.1 was tested in an experiment with 71 participants to obtain data suitable for analysis.

### 4.1. Data Collection

Video data and plant signals were collected during two sets of experiments performed in a controlled environment. The experiments were performed in accordance with the MIT Committee on the Use of Humans as Experimental Subjects (COUHES) guidelines.

In the first experiment, 40 individuals participated in the data collection, as described in Section 3.1. Due to some malfunctions in the plant sensor (no signal recorded) during the experiment, 12 data points had to be removed from the dataset, leading to 28 data points.

The second experiment was conducted on 31 individuals. As with the first experiment, an analysis of the quality of the data collected identified five incomplete data points, leaving 26 data points as input for the analysis.

The case study, therefore, focusses on the analysis of 54 data points using the methodology presented in Section 3.2.

### 4.2. Analysis

The data were analyzed using Python 3.8 and the available libraries, such as *keras* [39], *scipy* [40], *scikit-learn* [41], and *Numpy* [42]. The 54 data points from the video data and plant signals were used as input. Subsequently, normalization, partitioning, labeling, downsampling, and MFCC extraction were executed. The *dowsampling* hyperparameter was empirically set to 500 Hz. The video data were transformed into images with a frequency of 1 Hz. These images were labeled using *face-api.js* [22]. The following emotions were computed: Anger,Disgust,Fear,Happiness,Neutral,Sadness,Surprise.

Thus, the number of detectable emotions was $nb_{emotion}=7$, which was used for generating the models, as described in Table 2.

The approach described above applies to the first four models in our study, where windowing and downsampling techniques were utilized. In contrast, for the last three models (Random Forest, 1D CNN, and biLSTM), a different preprocessing strategy was employed. These models did not utilize downsampling due to their distinct handling of the raw data. Furthermore, during the analysis, it was observed that the classes of emotions were significantly unbalanced when no windowing preprocessing was carried out. Specifically, a large majority of instances were being classified as Neutral, leading to skewed results.

In order to address this imbalance and enhance the performance of the last three models, the Neutral emotion was excluded from the analysis. This adjustment resulted in a more balanced distribution of emotion classes and improved the models’ ability to distinguish between the remaining emotions. Consequently, for the Random Forest, 1D CNN, and biLSTM models, the set of emotions considered was reduced to six, namely Anger, Disgust, Fear, Happiness, Sadness, and Surprise. Therefore, in these cases, the number of detectable emotions was $nb_{emotion}=6$. This modification was crucial for ensuring the effectiveness and accuracy of these models in emotion detection tasks, as outlined in Table 2.

In order to optimize the other hyperparameters, grid searches were performed on the MIT SuperCloud high-performance compute cluster [43] using its GPU compute nodes with two Intel Xeon Gold 6248 20-core processors, 384 GB RAM, and two Nvidia Volta V100 GPUs with 32 GB VRAM each. A maximum of 50 epochs was used for the grid search. Table 4 shows the optimized parameters obtained by the grid-search algorithm for each model.

Once the hyperparameters were defined, model training was undertaken on the same MIT SuperCloud high-performance computing cluster that was used for the grid search. Final training was carried out over a maximum of 1000 epochs. The test set was then used to compute the overall accuracy (Equation (1)) and average recall per class (Equation (2)). In this study, the number of classes was either $nb_{class}=6$ or $nb_{class}=7$ since there are $nb_{emotion}=6$ or $nb_{emotion}=7$ emotions.

### 4.3. Evaluation

The results obtained for each of the models are presented in Table 5. Since five-split cross-validation was used, the values presented in Table 5 correspond to the average from the five splits.

**Figure 4:**
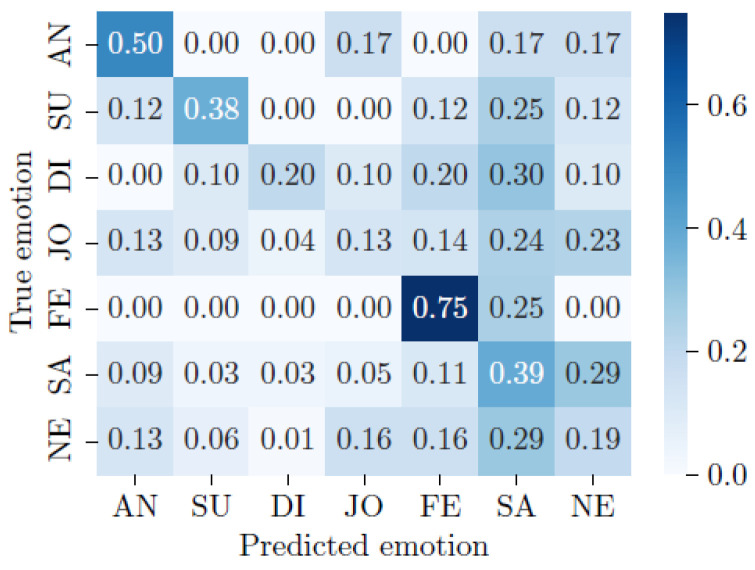
Confusion matrix of the final MFCC-ResNet plant classier, normalized per row for true emotion. The numbers represent the recall values. The labels represent the following emotions: AN = anger, SU = surprise, DI = disgust, JO = happiness, FE = fear, SA = sadness, and NE = neutral.

According to the results shown in Table 5, **MLP** gives the best accuracy ($Accuracy_{MLP}=0.399$) but also the worst recall ($Recall_{MLP}=0.220$). This is often a sign of an overfitting in the majority class. This behavior can also be observed for **MFCC-CNN** ($Accuracy_{MFCC-CNN}=0.377$ and $Recall_{MFCC-CNN}=0.275$). In the opposite case, **biLSTM** proposes a low $Accuracy_{biLSTM}=0.260$ but a relatively high $Recall_{biLSTM}=0.351$. Finally, **MFCC-ResNet** proposes the best balance between accuracy and recall with $Accuracy_{MFCC-ResNet}=0.318$ and $Recall_{MFCC-ResNet}=0.324$, respectively (see Figure 4).

**Figure 5:**
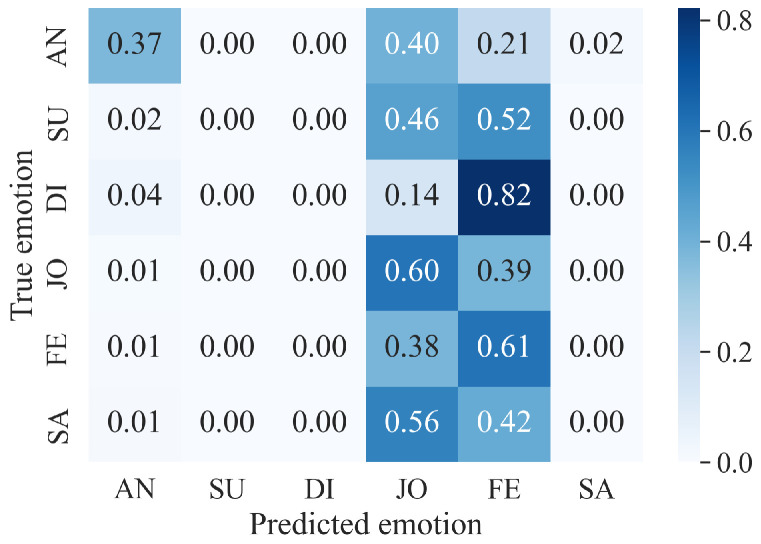
Confusion matrix of the final Random Forest (without windowing) plant classifier, normalized per row for true emotion. The numbers represent the recall values. The labels represent the following emotions: AN = anger, SU = surprise, DI = disgust, JO = happiness, FE = fear, and SA = sadness.

In contrast, the models utilizing the “raw data with no windowing” preprocessing approach show different performance characteristics. The **RF (no windowing)** model achieved the highest accuracy ($WeightedAccuracy_{RFnowindowing}=0.552$) and recall ($WeightedRecall_{RFnowindowing}=0.552$) among all models, indicating a strong overall performance. The **1D CNN (no windowing)** model also showed promising results, with an accuracy of $Accuracy_{1DCNNnowindowing}=0.461$ and a recall of $Recall_{1DCNNnowindowing}=0.514$, suggesting a good balance in its ability to correctly classify emotions. Lastly, the **biLSTM (no windowing)** model exhibited an accuracy of $Accuracy_{biLSTMnowindowing}=0.448$ and a recall of $Recall_{biLSTMnowindowing}=0.380$, which reflects its competent performance, although it slightly lags behind the RF and 1D CNN models in this specific setup (see Figure 5).

The confusion matrix resulting from the **MFCC-ResNet** model shows the model’s strong potential for detecting fear and anger, with 75% and 50% correctly predicted emotions for these two classes. Sadness is correctly detected in 39% of cases but can be mistaken for a neutral emotion, and fear is correctly detected in 29% and 11% of cases, respectively. Neutral, happiness, and disgust are difficult for the model to predict. The model’s performance for these classes is below 20%.

In comparison, the **RF (no windowing)** model shows varied performance across different emotions. For anger (AN), it has a recall of 0.373, a precision of 0.721, and an F1 score of 0.492. The model is unable to effectively detect surprise (SU), disgust (DI), and sadness (SA), with the recall, precision, and F1 score all being 0.000 for these emotions. Happiness (JO) and fear (FE) are better detected, with the model achieving a recall of 0.604 and 0.610, a precision of 0.556 and 0.540, and F1 scores of 0.579 and 0.573, respectively. This indicates that the model has a stronger ability to recognize emotions such as happiness and fear, whereas it struggles significantly in recognizing surprise, disgust, and sadness.

**Figure 6:**
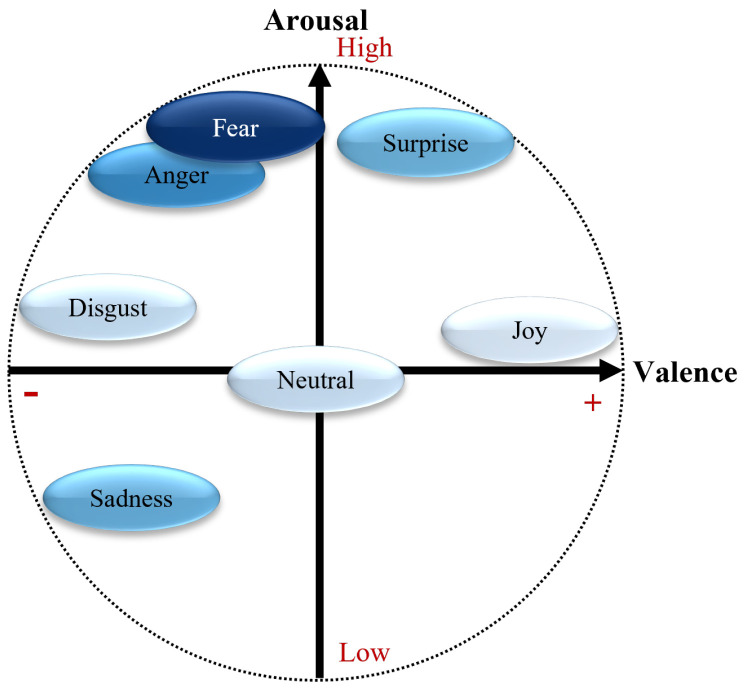
Confusion matrix results applied to the valence-arousal model.

These results can be explained using Figure 6, with the *valence-arousal* emotional model from Russell [20] and the basic emotions of Ekman [19] presented in Section 2.1.

As we can see in Figure 6, emotions with a high intensity of arousal, whether high or low, are relatively well-predicted by the **MFCC-ResNet** model. Fear and anger are two emotions with a high arousal value and a negative valence. These two emotions are recognized best by the model, with 75% and 50% correct classifications. For the **RF (no windowing)** model, anger shows a moderate performance, with a recall of 0.373 and an F1 score of 0.492, whereas fear is better recognized, with a recall of 0.610 and an F1 score of 0.573.

Sadness has a relatively low arousal value and negative valence. The **MFCC-ResNet** model’s performance for classifying this emotion is equivalent to that for detecting the emotion of surprise, which has high arousal but positive valence. The rate of correct detection of the two emotions, which are symmetrical with the neutral emotion in the valence-arousal conceptualization of emotions, is equivalent to around 38%. Both emotions are poorly predicted by the **RF (no windowing)** model, with the recall, precision, and F1 scores all being 0.000 for sadness and surprise.

Finally, emotions with a medium level of arousal, such as joy, disgust, and neutral emotions, are difficult for the **MFCC-ResNet** model to classify, with a correct classification rate of no more than 20%. However, joy shows a relatively better performance in the **RF (no windowing)** model compared to disgust, with a recall of 0.604 and an F1 score of 0.579.

Note that the accuracy of the **MFCC-ResNet model** is unweighted, which makes sense because the underrepresented categories have been oversampled during training to get a balanced distribution, as the original data distribution was as follows: anger: N = 6, surprise: N = 8, disgust: N = 10, joy: N = 95, fear: N = 4, sadness: N = 149, and neutral: N = 194. For the **RF (no windowing)** model, the unbalanced data were directly used for the model, which led to a higher *weighted* accuracy than that of the **MFCC-ResNet model**. The N, in this case, equates to the following: fear: 141, joy: 4927, surprise: 390, anger: 588, sadness: 4676, and disgust: 230.

**Figure 7:**
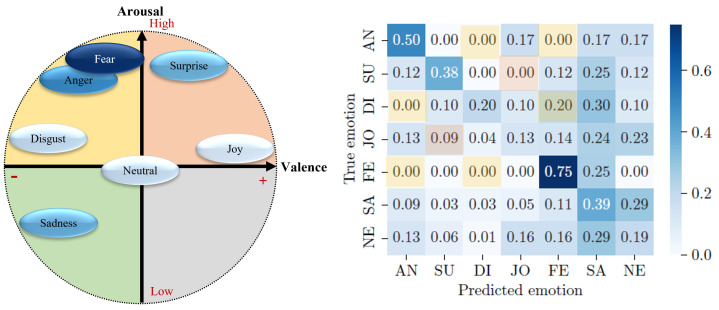
Intra-quadrant analysis of the confusion matrix based on the valence-arousal model. Colors in confusion matrix correspond to colors of quadrant in valence-arousal model.

Using the confusion matrix associated with the valence-arousal model of emotions also enables the analysis of the distribution of the model’s classification errors. Indeed, if the different emotions are grouped according to the quadrant to which they belong, as shown in Figure 7, one can see that the models rarely make classification errors within a single quadrant, but rather, they tend to decipher an emotion as belonging to another quadrant of the valence-arousal model.

The first frame, shown in orange in Figure 7, corresponds to emotions with positive valence and high arousal, i.e., surprise and joy. When surprise is detected, it is never confused with joy. Conversely, when joy is detected by the **MFCC-ResNet** model, it is confused with surprise in 9% of cases. Surprise is the second-least confused emotion (with joy) after disgust (4% confusion).

The **MFCC-ResNet** model’s ability to distinguish between emotions within the same quadrant is all the more apparent when the model’s second quadrant is studied since the frame represents a negative valence and a high arousal level, shown in yellow in Figure 7, no inter-class confusion is found, with the exception of the model’s classification of disgust as fear.

## 5. Discussion

Based on the results introduced above, the use of plants as sensors for emotion recognition deserves further investigation. The best model (**MFCC-ResNet**) shows an overall accuracy of nearly 32% and an average recall per class of 32.4%. The model is particularly good at recognizing emotions with high arousal levels and negative valences, with 75% and 50% accuracy for the fear and anger classes.

Furthermore, the **RF no windowing** model demonstrates even more promising results in this context. This model achieved an overall accuracy of 55.2% and displayed significant strengths in recognizing certain emotions. For instance, it had a high recall of 61.0% for fear, 60.4% for happiness, and 37.3% for anger, indicating its effectiveness in identifying these high-arousal, negative-valence emotions. However, it is important to note that the model struggled with emotions such as surprise, disgust, and sadness, as indicated by a recall and precision of 0.000 for these classes. This variation in performance across different emotions highlights the complexity of emotion recognition using plant signals and underscores the potential of further refining these models to improve their accuracy and recall across a broader spectrum of emotions.

This phenomenon might be explained by the type of sensor used to collect signals from the plant and the physiological responses associated with emotions. As described in Section 2.2, the *SpikerBox* sensor measures the potential difference between the plant and the soil. According to Rooney et al. [44], emotional arousal increases skin conductivity. Thus, the study of plants’ ability to detect variations in emotional arousal based on the electrical activity of individuals’ skin could be explored in future research.

Another hypothesis would explain the model’s performance by the physical reactions triggered by the respective emotions. Emotions such as fear, anger, or surprise lead to more body movements than emotions such as disgust or joy. The latter emotions result in small movements, often facial expressions, which only marginally influence the environment surrounding the plant. Thus, the strength of the physical reactions triggered by an emotion could explain the model’s recognition performance.

The fact that the emotion of sadness is also overused for the classification of other emotions also raises the question of the uniqueness of sadness. Although considered a basic emotion by [19], Shirai and Suzuki [45] argue that sadness is not unique and is more complex. “The exact nature of sadness is still quite vague in comparison to other emotions” [45]; this vagueness could be one of the explanations for the lack of precision observed in the classification of sadness. Since signal labeling is based on the recognition of emotions via images (see Section 3.3.2), a poor definition of the emotion of sadness can lead to poor signal labeling, resulting in model confusion for this class.

Nevertheless, these results also demonstrate that the deep learning tools typically used for multiple classification tasks, such as emotion recognition, can also be used to process signals from plants. As mentioned by He et al. [35], the depth of the CNN network does have a positive impact on model performance ($Recall_{MFCC-CNN}<Recall_{MFCC-ResNet}$). Moreover, taking into account the temporal aspect of signals improves model sensitivity ($Recall_{biLSTM}>Recall_{MLP}$). A relevant line of research would be to join these two models to make a hybrid model, utilizing the performance of the ResNet model while taking into account the temporality of signals with LSTMs. This combination has already been successfully used by Yu and Sun [46] in recognizing emotions from physiological data.

### Limitations and Further Research

It is important to underline certain limitations of the present study to identify relevant areas of research for future investigation.

First of all, data collection by the *SpikerBox* Brains [31] sensor allows for the acquisition of the plant’s electrical signals using external electrodes. However, the use of external electrodes can lead to inaccuracy in the collected signal [13]. The use of internal electrodes could be considered to improve the quality of the collected signal and quantify its influence on the plants’ ability to recognize emotions.

Moreover, the effectiveness of plant-based sensors has only been proven in extremely controlled environments [11,13,30]. This is also the case in this study. However, it is rare that the environment in which individuals operate on a daily basis is strictly controlled. Therefore, to propose real applications for emotion recognition based on plants, it would be interesting to test the model’s performance in a real-world environment and to carry out a sensitivity analysis of the model’s external influence factors.

Another limitation can be found in the data preparation phase. The proposed method uses the last image of a time window as a representation of the emotions felt during that period. This method enables rapid labeling but may limit the coherence of the emotion associated with the plant signal. However, this consistency of emotions over time is crucial for model learning. The labeling task could be improved by taking the class of the majority of emotions detected by the face-api interface over the period as a representation of the emotional state of the time window. Additionally, the use of another deep learning model for signal labeling (*face-api* [22]) might lead to an accumulation of errors. An estimate of this risk could be of interest to better analyze the results obtained by the plant-based emotion recognition model.

Moreover, as with any deep learning model, a lack of explicability and overfitting are two limitations of the proposed method. The presumed overfitting in the **MLP** and **MFCC-CNN** models is a risk that it is important to mitigate. The use of the *EarlyStopping* function and the optimization of hyperparameters by using a grid search help to limit it. However, overfitting remains an inherent limitation of deep learning models.

Finally, we need to address the issue of obtaining varying results based on the windowing and no windowing data preprocessing approaches.

In the domains of signal processing and time-series analysis, the choice of preprocessing techniques plays a crucial role in the performance of machine learning models. The observed variation in the results between the models using windowing and those employing a no-windowing approach can be attributed to fundamental differences in how these preprocessing strategies manipulate and represent the underlying data.

Windowing, a technique commonly used in time-series analysis, involves segmenting the signal into smaller, fixed-size segments or ‘windows’. This process enables the model to capture temporal dynamics and short-term patterns within each window, which can be crucial for understanding signals in time-dependent structures. By focusing on these localized segments, windowing can enhance the model’s ability to detect subtle changes and temporal patterns that might be indicative of specific emotional states. Moreover, windowing can also help reduce noise and manage computational complexity by simplifying the data structure.

On the other hand, the no-windowing approach processes the signal in its entirety or in larger segments. This method preserves the global context and long-range dependencies in the data, which can be particularly beneficial for capturing overall trends and patterns across the entire signal. However, this approach may overlook finer, localized temporal features that are critical for distinguishing between certain emotional states. The larger data segments also increase the complexity of the model, which can lead to challenges in learning and generalization, especially when dealing with high-dimensional data.

Furthermore, the inherent characteristics of the plant signals being analyzed also contribute to the differential performance of these models. Plant-based bio-signals might exhibit variations in both short-term and long-term patterns when responding to emotional stimuli. Therefore, models employing windowing may be better suited to capture rapid, transient responses, while no windowing models might be more effective in detecting sustained or cumulative signal responses over time.

Therefore, the choice between windowing and no windowing approaches reflects a trade-off between capturing localized temporal features and preserving global signal characteristics. The effectiveness of each method is contingent upon the nature of the data and the specific requirements of the emotion detection task. This underscores the importance of selecting an appropriate preprocessing strategy in signal-based emotion recognition, especially in novel and complex domains such as plant signal analysis.

This research opens the way to emotion recognition using non-intrusive elements such as plants. Other research, such as the use of a continuous paradigm [20] to carry out regression on plant signals, the use of the inter-class confusion of the model to better understand the links between emotions, and the use of personal characteristics such as personality traits as dependent variables in recognizing emotions, should be investigated to improve the proposed methodology.

## 6. Conclusions

The recognition of human emotions is a popular research topic in behavioral science. Several conceptualizations have made it possible to structure emotions discretely [19] or continuously [20] from a behavioral point of view. Current technologies enable emotion recognition from audio recordings [6,7], video recordings [3,4,5], text [8,9,10], and even physiological data [24]. However, these data sources are expensive, intrusive, and regulated [11]. This is why it is important to find new sources of information that can monitor people’s emotions without bothering them. In this paper, we introduce a novel method that uses plants as biosensors to measure human emotions. As plants have no privacy concerns, using them as sensors provides a privacy-respecting way of measuring human emotions, opening up new ways of using emotion recognition in environments sensitive to privacy in public spaces such as supermarkets and museums. Even more, using plants as sensors to measure human emotions will increase human well-being, as interaction with nature and plants is good for all aspects of human health and will increase creative and cognitive performance [47].

Plants do not just have amazing abilities to recognize chemical elements in their environment [30]; they are able to detect sounds [13], people, and moods [11]. For these reasons, a four-step methodology enabling the use of plants as human emotion detectors has been introduced. First, the data were prepared by denoising, formatting, and labeling the plant signals using video data. Then, different machine learning and deep learning models (MLP, biLSTM, MFCC-CNN, MFCC-ResNet, Random Forest, and 1D CNN) were created and parameterized using the cross-validation and grid-search algorithms to optimize the parameterization for each model. The best models were trained and used for classification. The detection of an emotion based on only a plant sensor is the result of this method. This study demonstrates that plants might, indeed, be used to measure human emotions, opening up new areas of research as well as new areas of practical application for this technology. For instance, plant-based sensors could also be used to distinguish between different persons, providing a non-intrusive way of access control, thus extending earlier research by Oezkaya and Gloor [11]. In ongoing research, our team is currently investigating the capability of plants to identify different types of body movements, for instance, distinguishing arm movement from leg movement, further exploring the fine-grained sensitivity of plants.

In view of the results presented in Section 4, the hypothesis that plants can be used for the recognition of emotions is worth investigating further. However, it is important to note that the use of external electrodes, the need for a controlled environment, the labeling process based on the last emotion felt, the lack of applicability, and the overfitting of models remain limitations in this type of study. However, these limitations can also be seen as fantastic research opportunities that should be explored in future work. Other avenues of research, such as the use of the continuous paradigm [20] to regress by using plant signals, the use of model inter-class confusion to better understand the links between emotions, and the use of personal characteristics, such as personality traits, as a dependent variable to recognize emotion, should also be investigated.

The proposed methodology is a first step toward an innovative sensor that will address the concerns and high costs of traditional sensors and pave the way for new areas of research in human emotion detection and recognition.

## Figures and Tables

**Table 1:** Description of the video sequence used to elicit participants’ emotions (adapted from [32]).

Video ID	Name	Short Description	Expected Emotion	Duration (s)
**1**	Puppies	Cute puppies running	Happiness	13
**2**	Avocado	A toddler holding an avocado	Happiness	8
**3**	Runner	Competitive runners supporting a girl from another team over the finish line	Happiness	24
**4**	Maggot	A man eating a maggot	Disgust	37
**5**	Raccoon	Man beating raccoon to death	Anger	16
**6**	Trump	Donald Trump talking about foreigners	Anger	52
**7**	Montain bike	Mountain biker riding down a rock bridge	Surprise	29
**8**	Roof run	Runner almost falling of a skyscraper	Surprise	18
**9**	Abandoned	Social worker feeding a starved toddler	Sadness	64
**10**	Waste	Residents collecting electronic waste in the slums of Accra	Sadness	31
**11**	Dog	Sad dog on the gravestone of his master	Sadness	11
**12**	Roof bike	Person biking on top of a skyscraper	Fear	28
**13**	Monster	A man discovering a monster through his camera	Fear	156
**14**	Condom ad	Child throwing a tantrum in a supermarket	Multiple	38
**15**	Soldier	Soldiers in battle	Multiple	35

**Table 2:** Synthesis of the architectures of the models.

Model Name	Utility	Input	Architecture
**MLP**	Baseline	Downsampled plant signal	Alternation of ReLu-activated densely connected layers with dropout layers to limit overfitting. The last layer is a SoftMax-activated dense layer of $nb_{emotion}$ neurons.
**biLSTM**	Considers the temporal dependencies of the plant signal		Two blocks’ model LSTM Layers embedded in a bi-directional wrapper Alternation of two ReLu-activated dense layers with dropout layers. Each dense layer is composed of 1024 and 512 neurons respectively. The last layer is a SoftMax-activated dense layer of $nb_{emotion}$ neurons.
**MFCC-CNN**	Specialized in 2D or 3D inputs, as in multifeatured time-series	MFCCs features	Two blocks’ model. Alternation of convolutional layers with 2×2 max pooling operations. Alternation of ReLu-activated dense layers with dropout layers. The last layer is a SoftMax-activated dense layer of $nb_{emotion}$ neurons
**MFCC-ResNet**	Pretrained DeepCNN to emphasize the importance of the network depth		ResNet architecture slightly modified to fit the emotion detection task. The top dense layers used for classification are replaced by a dense layer of 1024 neurons, followed by a dropout layer. The last layer is a SoftMax-activated dense layer of nbemotion neurons
**Random Forest not windowed**	Effective for diverse datasets. Good overall robustness.	Raw plant signal normalized, not windowed	Utilizes an ensemble of decision trees. Parameters include $n_{estimators}$: 300 (number of trees), $\max_{depth}$: 20 (maximum depth of each tree), and ${\mathrm{class}}_{weight}$: None. This configuration is aimed at handling complex classification tasks, balancing bias and variance.
**1-Dimensional CNN not windowed**	Suitable for time series analysis		Sequential model with a 1D convolutional layer (64 filters, kernel size of 3, ‘swish’ activation, input shape of (10,000, 1)). Followed by a MaxPooling layer (pool size of 2), a Flatten layer, a dense layer (100 neurons, ‘swish’ activation), and an output dense layer (number of neurons equal to unique classes in ‘y’, ‘softmax’ activation). Compiled with Adam optimizer, ‘${\mathrm{sparse}}_{categorical\_crossentropy}$’ loss, and accuracy metrics. The last layer is a SoftMax-activated dense layer of nbemotion neurons
**biLSTM not windowed**	Considers the temporal dependencies of the plant signal		Sequential model with a Bidirectional LSTM layer (1024 units, return sequences true, input shape based on reshaped training data), followed by another Bidirectional LSTM layer (1024 units). Concludes with a dense layer (100 neurons, ‘swish’ activation) and an output dense layer (number of neurons equal to unique classes in ‘y’, ‘softmax’ activation). Optimized with Adam (learning rate 0.0003), using ${\mathrm{sparse}}_{categorical\_crossentropy}$ loss and accuracy metrics. The last layer is a SoftMax-activated dense layer of nbemotion neurons

**Table 3:** Synthesis of the hyperparameters to be tested by the Grid Search algorithm.

Model Name	Parameter	Values	Number of Configurations
**MLP**	dense Units dense layers Dropout Rate Learning Rate Balancing Window Hop	1024, 4096 2, 4 0, 0.2 3 × $10^{-4}$, 1 × $10^{-3}$ Balance, Weights, None 5, 10, 20 5, 10	288
**biLSTM**	LSTM Units LSTM layers Dropout Rate Learning Rate Balancing Window Hop	64, 256, 1024 1, 2, 3 0, 0.2 3 × $10^{-4}$, 1 × $10^{-3}$ Balance, Weights, None 5, 10, 20 5, 10	648
**MFCC-CNN**	Conv Filters Conv layers Conv Kernel Size Dropout Rate Learning Rate Balancing Window Hop	64, 128 2, 3 3, 5, 7 0, 0.2 3 × $10^{-4}$, 1 × $10^{-3}$ Balance, Weights, None 5, 10, 20 5, 10	864
**MFCC-ResNet**	Pretrained Number of MFCCs Dropout Rate Learning Rate Balancing Window Hop	Yes, No 20, 40, 60 0, 0.2 3 × $10^{-4}$, 1 × $10^{-3}$ Balance, Weights, None 5, 10, 20 5, 10	432
**RF no windowing**	Number of estimators Max Depth Balancing	100, 200, 300, 500, 700 None, 10, 20, 30 Balance, Weights, None	60
**1D CNN no windowing**	Conv Filters Conv layers Conv Kernel Size Dropout Rate Learning Rate Balancing	64, 128 2, 3 3, 5, 7 0, 0.2 3 × $10^{-4}$, 1 × $10^{-3}$ Balance, Weights, None	144
**biLSTM no windowing**	LSTM Units LSTM layers Dropout Rate Learning Rate Balancing	64, 256, 1024 1, 2, 3 0, 0.2 3 × $10^{-4}$, 1 × $10^{-3}$ Balance, Weights, None	108

**Table 4:** Synthesis of the optimized hyperparameters.

Model Name	Parameters	Values
**MLP**	Dense Units	4096
	Dense Layers	2
	Dropout Rate	0.2
	Learning Rate	0.001
	Balancing	Balanced
	Window	20 s
	Hop	10 s
**biLSTM**	LSTM Units	1024
	LSTM Layers	2
	Dropout Rate	0
	Learning Rate	0.0003
	Balancing	Balanced
	Window	20 s
	Hop	10 s
**MFCC-CNN**	Conv Filters	96
	Conv Layers	2
	Conv Kernel Size	7
	Dropout Rate	0.2
	Learning Rate	0.0003
	Balancing	Balanced
	Window	20 s
	Hop	10 s
**MFCC-ResNet**	Pretrained	No
	Number of MFCCs	60
	Dropout Rate	0.2
	Learning Rate	0.001
	Balancing	Balanced
	Window	20 s
	Hop	10 s
**RF no windowing**	Number of Estimators	300
	Max Depth	20
	Balancing	None
**1D CNN no windowing**	Conv Filters	96
	Conv Layers	2
	Conv Kernel Size	7
	Dropout Rate	0.2
	Learning Rate	0.0003
	Balancing	None
**biLSTM no windowing**	LSTM Units	1024
	LSTM Layers	2
	Dropout Rate	0
	Learning Rate	0.0003
	Balancing	None

**Table 5:** Final performance of all model architectures for the plant emotion data.

Model	Test Set Accuracy	Test Set Recall
**MLP**	0.399	0.220
**biLSTM**	0.260	0.351
**MFCC-CNN**	0.377	0.275
**MFCC-RestNet**	0.318	0.324
**RF (no windowing)**	0.552	0.552
**1D CNN (no windowing)**	0.461	0.514
**biLSTM (no windowing)**	0.448	0.380

## Data Availability

Data is available at request from the corresponding author.
